# Individual Factors Associated With COVID-19 Infection: A Machine Learning Study

**DOI:** 10.3389/fpubh.2022.912099

**Published:** 2022-06-30

**Authors:** Tania Ramírez-del Real, Mireya Martínez-García, Manlio F. Márquez, Laura López-Trejo, Guadalupe Gutiérrez-Esparza, Enrique Hernández-Lemus

**Affiliations:** ^1^Cátedras Conacyt, National Council on Science and Technology, Mexico City, Mexico; ^2^Center for Research in Geospatial Information Sciences, Mexico City, Mexico; ^3^Clinical Research Division, National Institute of Cardiology “Ignacio Chávez”, Mexico City, Mexico; ^4^Institute for Security and Social Services of State Workers, Mexico City, Mexico; ^5^Computational Genomics Division, National Institute of Genomic Medicine, Mexico City, Mexico; ^6^Center for Complexity Sciences, Universidad Nacional Autónoma de México, Mexico City, Mexico

**Keywords:** COVID-19, machine learning, feature selection, imbalanced data, predictive model

## Abstract

The fast, exponential increase of COVID-19 infections and their catastrophic effects on patients' health have required the development of tools that support health systems in the quick and efficient diagnosis and prognosis of this disease. In this context, the present study aims to identify the potential factors associated with COVID-19 infections, applying machine learning techniques, particularly random forest, chi-squared, xgboost, and rpart for feature selection; ROSE and SMOTE were used as resampling methods due to the existence of class imbalance. Similarly, machine and deep learning algorithms such as support vector machines, C4.5, random forest, rpart, and deep neural networks were explored during the train/test phase to select the best prediction model. The dataset used in this study contains clinical data, anthropometric measurements, and other health parameters related to smoking habits, alcohol consumption, quality of sleep, physical activity, and health status during confinement due to the pandemic associated with COVID-19. The results showed that the XGBoost model got the best features associated with COVID-19 infection, and random forest approximated the best predictive model with a balanced accuracy of 90.41% using SMOTE as a resampling technique. The model with the best performance provides a tool to help prevent contracting SARS-CoV-2 since the variables with the highest risk factor are detected, and some of them are, to a certain extent controllable.

## 1. Introduction

The exponential growth of infections by COVID-19, a disease associated with the SARS-CoV-2 virus leads to a global death burden, impelling the World Health Organization (WHO) to declare it a global pandemic (1). The virus can spread from an infected COVID-19 person to a healthy person through physical contact, mucous contact, or airborne transmission (2). It can be transmitted before starting showing symptoms or without ever developing symptoms at all. The COVID-19 pandemic has wreaked havoc globally, causing an economic crisis, a sanitary emergency, and confinement periods that affected people's lifestyles, habits, and daily activities (3).

Despite scientific advances in medicine, particularly the development of vaccines and Reverse Transcription Polymerase Chain Reaction (RT-PCR) tests to detect COVID-19, the pandemic has not been adequately controlled yet (3, 4). A timely and effective diagnosis remains crucial to save lives and prevent the spread of infections. Machine learning, an integral part of artificial intelligence, has been widely applied to predict or diagnose diseases, improve treatment accuracy, detect anomalies, and provide solutions to other aspects derived from the healthcare domain (5).

Concerning COVID-19, machine learning models have been developed to predict the risk of contracting the virus, indicating the severity, the risk of death, and other predictive tasks with great potential (6, 7). The timely and effective detection of COVID-19 has become an essential task for healthcare organizations since it may help decrease the deadly effect of the virus and support the planning of care (8–10). In these cases, machine learning models have been developed to assess the prognosis or mortality risk in patients with COVID-19 (11), for instance, used a Random Forest (RF) model to predict the forecasts of patients with COVID-19; similarly, the Gini index was used to identify the most critical variables (features) to assess risk and indicate the prognoses of patients.

The study by Pourhomayoun and Shakibi (12) included a dataset of 32 items related to demographic, physiological, and laboratory data and developed a predictive model to determine the health risk and also forecast the risk of mortality for patients with COVID-19. The techniques used there were: Support Vector Machines (SVM), Artificial Neural Networks (ANN), RF, Decision Tree (DTs), Logistic Regression, and K-Nearest Neighbor clustering (KNN). The ANN demonstrated the best performance with an accuracy of 93.75%.

Further research (13) has made use of computational intelligence methods to predict the daily total COVID-19 infections and deaths as observed during three lockdown schemas (partial, herd, complete). The techniques used were RF, K-NN, SVM, DTs, polynomial regression, Holt winter, ARIMA, and SARIMA. Finally, the authors concluded that herd lockdown is the best policy to control COVID-19.

In García-Ordás et al. (14), the authors studied the impact between the nutrition of the different countries and the number of deaths caused by COVID-19. They made clusters with K-means by country according to the distribution of fat, energy, and protein in 23 different types of food and the ingested in kilograms. They found a relationship between high-fat consumption and the highest death rates.

The study by Kenneth and So (15) presents the application of an extreme gradient boosting algorithm (XGboost) to predict mortality (AUC of 81.4%) and severity (72.3%) among infected individuals. The authors used 97 clinical features, specifically: demographic variables, comorbidities, blood measurements, anthropometric measures, and other risk factors (e.g., smoking/drinking habits).

The analysis by Sun et al. (16) also used XGboost to predict COVID-19 severities achieving a mean micro-average AUROC (area under the receiver operating characteristic curve) of 97%. Moreover, a mean micro-average AUPR (area under the precision-recall curve) of 94%, using 60 features (consisting of 19 proteins, 11 metabolites, seven lipids, and 23 mRNAs) was also achieved.

In García-Ordás et al. (14), the authors studied the association between the feed habits of the diverse nations and the number of deaths caused by the illness. The authors used demographic, clinical, physiological, and biochemical tests. The authors proposed an application to detect critical features and faculties of self-care in individuals with COVID-19 disease, and infectious and internal medicine specialists selected the elements to consider in self-monitoring. They concluded that interventions encouraging healthy conduct are essential conditions of COVID surveillance (17).

However, other known risk factors for illness and death from COVID-19, associated with sleep disturbances, physical activity, alcohol, metabolic syndrome, and poor diet were not included in their analysis (18–22). In this stdy, we used a dataset related to clinical and anthropometric parameters, biochemical screening, sleep disturbances, physical activity, alcohol, diet, habits, and health status during the confinement due to the COVID-19 pandemic (refer to Table 1). The primary purpose is to identify the main features of the participants who contracted COVID-19, based on their health history as registered and stored in the Tlalpan 2020 project (23), and considering the follow-up questionnaire to determine the most importable risk factors for infection.

**Table 1:** Dataset variables.

**Variable**	**Name**	**Type**
Age	Age	Numeric
Sex	Sex	Dichotomous
weight	Weight	Numeric
height	Height	Numeric
BMI	Body mass index	Numeric
waist	Waist circumference	Numeric
SBP	Systolic blood pressure	Numeric
DBP	Diastolic blood pressure	Numeric
Phyactmet	Physical activity measured in metabolic Equivalent of task (METs)	Dichotomous
anxst	State Anxiety	Factor: range from 1 to 4
anxtr	Trait anxiety	Factor: range from 1 to 4
slpsnrr1	Snoring during sleep	Factor: range from 1 to 5
slpsob1	Sleep short of breath or headache	Factor: range from 1 to 5
slps3	Sleep somnolence	Factor: range from 1 to 5
slpop1	Optimal Sleep	Dichotomous
smk	Smoking habit	Dichotomous
EtOH_avg	Frequency alcohol consumption	Dichotomous
uric	Uric acid	Numeric
crea	Creatinine	Numeric
HDL	High-density lipoprotein	Numeric
LDL	Low-density lipoprotein	Numeric
glu	Glucose	Numeric
chol	Cholesterol	Numeric
trig	Triglycerides	Numeric
na1	Serum sodium	Numeric
met_s	Metabolic syndrome	Dichotomous
wrk_f	Outdoor work	Dichotomous
wrk_h	Home office	Dichotomous
umplyd	Unemployed	Dichotomous
wrk_hsp	Working in hospital	Dichotomous
wrk_off	Working in office	Dichotomous
MaritStat	Marital status (single or married)	Dichotomous
cocr	Worry for contagion of the COVID-19	Factor: range from 0 to 2
trbslpt	Sleep problems during COVID-19 pandemic	Dichotomous
quislt	Isolation during COVID-19 pandemic	Factor: range from 0 to 4
outli	Outings limited during COVID-19 pandemic	Dichotomous
kpgoing	Keep coming out with precautionary measures	Dichotomous
phyact	Physical activity during the pandemic	Factor: range from 0 to 4
violence	Domestic violence during pandemic	Dichotomous
EtOH_q	Frequency alcohol consumption during pandemic	dichotomous
obsty	Obesity	Numeric
ovrw	Overweight	Numeric
smk_q	Smoking during pandemic	Dichotomous
anxdsr	Anxiety during pandemic	Dichotomous
hipert	Hypertension during pandemic	Dichotomous
news_f	Listen to the news by the family	Dichotomous
news_sn	See to the news by social networks	Dichotomous
news_tv	Listen to the news on the television or radio	dichotomous
lckd_hosp	Hospitalization for COVID-19 infection	Dichotomous
COVID	Diagnosis of COVID-19	Dichotomous

*anxst is 1 = not at all, 2 = a little, 3 = quite, 4 = a lot*.

*anxtr is 1 = rarely, 2 = sometimes, 3 = frequently, 4 = usually*.

*slpsnrr1 is 1 = 100, 2 = 80, 3 = 60, 4 = 40, 5 = 20, 6 = 0, being the value of 100, the bigger problem*.

*slpsobl is 1 = 100, 2 = 80, 3 = 60, 4 = 40, 5 = 20, 6 = 0, being the value of 100, the bigger problem*.

*slps3 is 1 = 100, 2 = 80, 3 = 60, 4 = 40, 5 = 20, 6 = 0, being the value of 100, the bigger problem*.

*cocr is 0 = not at all, 1 = a little, 2 = quite, 4 = a lot*.

*quislt is 1 = not at all, 2 = a little, 3 = quite, 4 = a lot*.

*phyact is 1 = not at all, 2 = a little, 3 = quite, 4 = a lot*.

Identifying potentially modifiable lifestyle and risk factors increasing the odds of infection during a novel pandemic (such as COVID-19) is highly relevant since it will provide the health policy authorities with further information to broaden the spectrum of non-pharmacological interventions (NPI), perhaps to include data-driven strategies to lower population risks (24–27). NPIs are still relevant to preventing infections, despite the advancement of population-level vaccination (and in the absence of widespread targeted therapies to treat people already infected); in particular, in the context of the surge of new SARS-CoV2 variants, some of which may potentially escape the effects of current vaccines.

Indeed, the use of computational intelligence and data analytics approaches for the vigilance and early survey of SARS-CoV2 infection has been an extremely relevant topic during the COVID-19 pandemic. Shabbir and collaborators (28) have implemented a strategy based on exploratory data analytics from diverse sources, coupled with telemonitoring and the use of internet of things (29–31) to detect COVID-19 severity in the context of *smart hospitals* (32–34). Also relevant is the use of concepts from computational social science (ambient intelligence, in particular) and again data from wearables (in this case, smartwatches) to develop early warning alerts (35–37). Several additional approaches to use machine learning to prevent or warn in advance for COVID-19 are discussed in the monographic review by Saeed et al. (38). The authors present a survey of recent literature regarding invasive non-invasive or non-contact technologies to detect, diagnose, and monitor human activities (39–41), particularly those inducing risks for COVID-19 infection or reflecting individuals with related symptoms, such as irregular respiration, in an automated fashion. Additional advances along these lines can be found in the studies by Kallel et al. (42), Conroy et al. (43), Pandey et al. (44), and Khoa et al. (45), to name but a few remarkable studies.

Despite all these timely and worthy contributions, much of these require special efforts, measurement devices, and infrastructure that may not be available at a large scale in under-developed or in-development economies. Even in medium-to-high income countries such as Mexico and even in the context of a large metropolis such as Mexico City there are large disparities in health services that prevent such (somehow sophisticated) strategies to be applied massively. In this regard, the contributions of this study will be centered on providing a machine learning approach to analyze relatively accessible clinical and sociodemographic data available in most medium-to-large hospitals (i.e., those that can treat most COVID-19 hospitalized cases), in order to provide clues for health officials to monitor for risk factors in large populations. The conditions needed for our analyses are thus of more broad applicability, in particular in places with disparities in access to healthcare services and appliances.

This article is organized as follows: In Section 2, the materials and methods are introduced. In Section 3, computational experiments' performance is shown and results are presented. A discussion (Section 4) and some concluding remarks are given (Section 5), also some ideas on the implications for future studies are outlined.

## 2. Materials and Methods

### 2.1. Data

The dataset comprised in this research was acquired from the Tlalpan 2020 study (23), a cohort at the National Institute of Cardiology in Mexico (Instituto Nacional de Cardiología-Ignacio Chávez, INC-ICh) [IRB approval code 13-802]. Data was collected from the baseline of 714 healthy adult residents of Mexico City between 20 and 50 years old. Also, a follow-up survey to know participants' habits and health status during confinement due to the COVID-19 pandemic; a total of 218 participants confirmed having contracted the COVID-19 infection. It is essential to mention that all participants gave written informed consent.

This dataset includes health variables that are related to anthropometric measurements and clinical parameters, biomedical tests, other factors such as smoking habit, alcohol consumption, physical activity, psychological stress level, sleep disorders, dietary as well as habits, and health status during the confinement due to pandemic associated with COVID-19 (refer to Table 1). Also, it is essential to mention that the dataset is imbalanced; this scenario is expected in medical diagnoses for detecting illnesses (46).

#### 2.1.1. Anthropometric Measurements and Clinical Parameters

The International Society for the Advancement of Kinanthropometry (ISAK) policies (47) declare necessary measurements with the patient fasting, particularly the weight, height, and waist circumference. The ratio between weight and height to the square is the BMI, and the ratio of waist and height is the WHtR in cm. Another registration is the blood pressure, specifically systolic (SBP) and diastolic (DBP); therefore, the record consists of the average of three measures with a 3-min gap. The JNC7 standard procedure defines the hypertension status when SBP ≥ 140 mm Hg, a DBP ≥ 90 mm Hg, or both (48).

#### 2.1.2. Biochemical Tests

The records for the screen test consist of measuring fasting plasma glucose (FPG), triglycerides (TGs), and high-density lipoprotein-cholesterol (HDL-C) in blood after 12 h of overnight fasting at the Central Laboratory of INC-ICh.

#### 2.1.3. Additional Risk Factors

(1) The classification for the smoking practice is as a never, retired or present smoker.
(2) In the case of alcohol consumption, the category is a present drinker or not; the number of drinks (cups or beers) and frequency is another registration.
(3) The extended version of the International Physical Activity Questionnaire, IPAQ (49) measures the physical conditioning, through the activity in METs (metabolic equivalents)-minutes/week, and the categories are low, moderate, and high, *via* questions concerning four occupations: work, home, transportation, and leisure time.
(4) Psychological stress level was determined by the State-Trait Anxiety Inventory (STAI) categorized into five categories high-level anxiety (>65), moderate-high anxiety (56–65), medium anxiety (46–55), minor anxiety (36–45), and low-level anxiety (<35) (50, 51).

In the case of (5) sleep disorders, the Medical Outcomes Study-Sleep scale of 12 items was measured (52, 53).

#### 2.1.4. Habits and Health Status During the Confinement Due to the COVID-19 Pandemic

The following habits and health status during confinement due to the COVID-19 pandemic were collected.

(1) Workplace during pandemic (wrk_f, wrk_h), (2) Degree of concern about COVID-19 (cocr), (3) Isolation level during the pandemic (quislt, outli, kpgoing), (4) Diseases and comorbidities, (5) Situations of family violence during the pandemic (violence), (6) Media consulted for news about the pandemic (news_f, news_sn, and news_tv), (7) Diagnosis of COVID-19 (COVID), (8) Recovery place COVID-19 (lckd_hosp), (9) Cigarettes consumed per day (smk_q), and (10) Diagnosis of hypertension (hipert) and (11) physical activity during the pandemic (phyact), which was defined by exercising at least three times per week for at least 30 min per session according to the minimum guidelines by American College of Sports Medicine (54).

### 2.2. Methods

**Figure 1:**
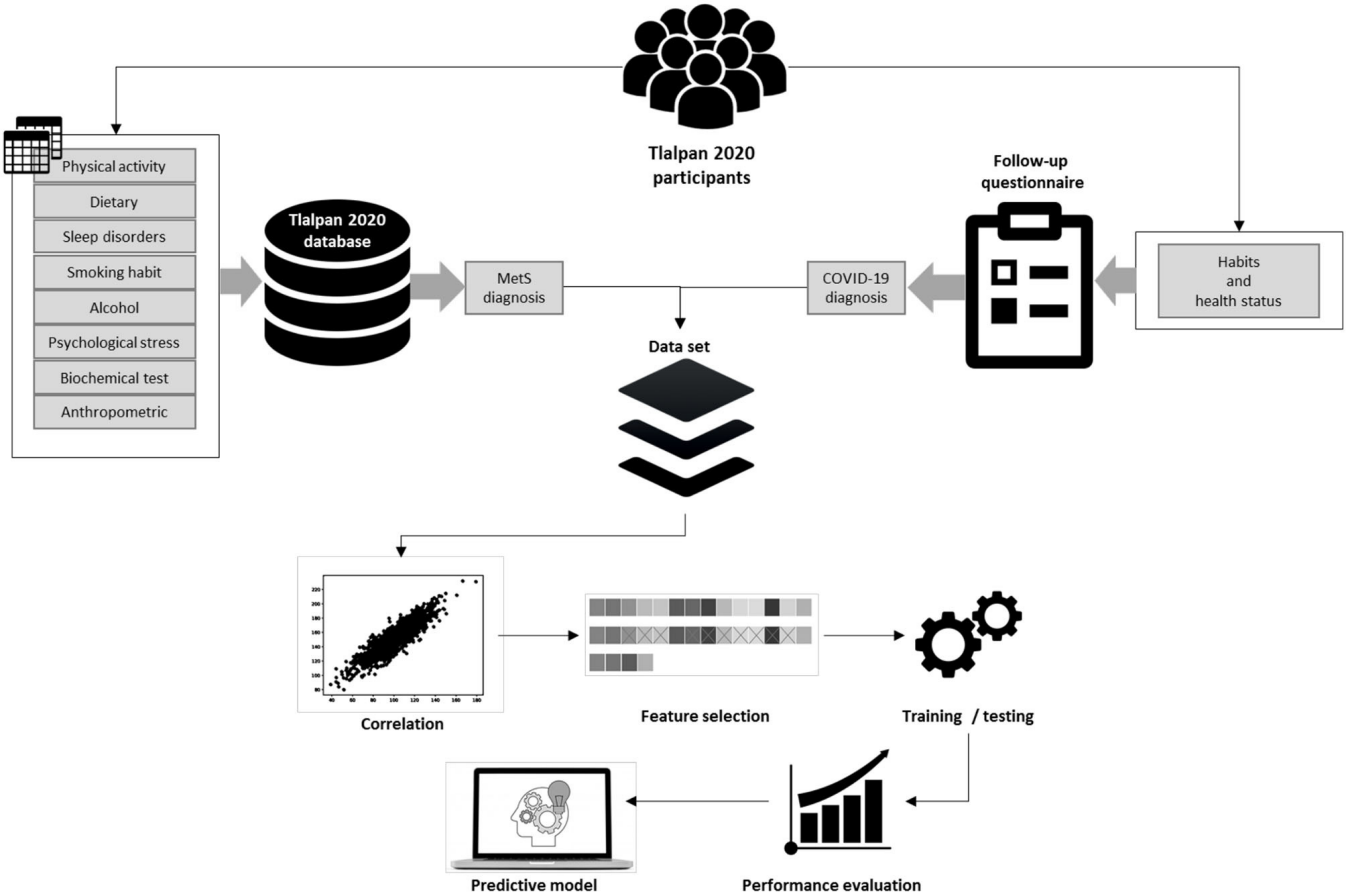
Prediction model.

Figure 1 illustrates a general representation of the prediction model and describes the methodology applied, where we used the data that the participants have provided to the Tlalpan 2020 project (physical activity, dietary, sleep disorders, smoking habit, alcohol consumption, psychological stress, biochemical test, and anthropometric) in visits and follow-ups, as well as the data from the follow-up questionnaire carried out during the COVID-19 lockdown.

Moreover, we used the National Cholesterol Education Program Adult Treatment Panel III criteria to classify participants with metabolic syndrome (MetS). From the follow-up questionnaire, it was possible to extract the habits and health status and positive COVID-19 infections from the same participants.

Once the dataset was conformed, we applied feature selection methods (Chi-squared, random forest, rpart, and Xgboost) to obtain the essential variables. Subsequently, we performed a correlation coefficient analysis to determine irrelevant and redundant features to create a new subset of features that contains the best features obtained by each method. The dataset was divided into two-thirds for the training and one-third for testing. Consequently, we applied data balancing methods such as over-sampling, under-sampling, and synthetic minority oversampling technique (SMOTE) to change the class distribution in the training dataset.

In this study, we applied four machine learning models: random forest, CART, C4.5, XGBoost, as well as deep neural networks, based on their high performance to diagnose COVID-19 (11, 55–57). To evaluate each model we made 30 executions with different seeds. Subsequently, we evaluated the models based on the following performance measures: sensitivity (SENS), specificity (SPC), accuracy (ACC), balanced accuracy (B.ACC), and the geometric mean (G-means); these last two metrics have been used for imbalanced data learning assessment (58). Finally, an optimized predictive model was obtained.

#### 2.2.1. Data-Balancing Methods

The data-balancing methods improve the performance of machine learning models when the class distribution in a dataset is not equal. Models have a better performance in the majority class and a higher misclassification rate in the minority class (59). For this reason, we used two-hybrid methods, the function Random Over-Sampling Examples (ROSE) from the ROSE package (60) and SMOTE from performanceEstimation package (61), to change the class distribution in the training dataset.

Datasets related to COVID-19 have imbalanced data (62); some studies declare the improvement of machine learning methods applying SMOTE technique (63–66) and a novel variant of SMOTE (67), also ROSE is used (68).

#### 2.2.2. Correlation Coefficient Analysis

The correlation coefficient analysis allows the feature selection procedure to measure the relationship between the dataset variables. The range of correlation values is between -1 and 1, indicating the relationship's dependency on the variables. To make this process, we used Pearson correlation, with a correlation coefficient threshold of 0.5, as defined by Equation 1 (69):


(1) $$\begin{array}{l} \begin{array}{c} pcc(u,u^{\prime })=\frac{\sum_{i\in I}\left(r_{u,i}-{\bar{r}}_{u})(r_{u^{\prime },i}-{\bar{r}}_{u^{\prime }}\right)}{\sqrt{\sum_{i\in I}{\left(r_{u,i}-{\bar{r}}_{u}\right)}^{2}}\sqrt{\sum_{i\in I}{\left(r_{u,i}-{\bar{r}}_{u^{\prime }}\right)}^{2}}}, \end{array} \end{array}$$

where *r*_*u, i*_ and $r_{u^{\prime },i}$ are the contribution scores, and also ${\bar{r}}_{u}$ and ${\bar{r}}_{u^{\prime }}$ are the average assortments.

#### 2.2.3. Chi-Square

Chi-square is a statistical test –based on the eponymous statistic and distribution– commonly used in machine learning to rank variables and support the feature selection process (70). Given a feature *f* and the class *c* ($\bar{f}$, $\bar{c}$ as complements), the chi-squared could be computed as follows:


(2) $$\begin{array}{l} X^{2}=\overset{k}{\underset{i=1}{\sum }}\frac{{\left(x_{i}-m_{i}\right)}^{2}}{m_{i}} \end{array}$$

where *k* is the number of classes, *x*_*i*_ is the frequency of occurrence in class *i*, and the *m*_*i*_ is the expected frequency for the same class.

#### 2.2.4. ANOVA

Another method used to rank the importance of continuous variables was the analysis of variance (ANOVA), which is a family of statistic tests applied to compare if the means of two or more samples are significantly different. ANOVA tests can be implemented for feature selection (71), in this study, we used ANOVA f-tests to estimate the ranks of features.

#### 2.2.5. Random Forest

Random forest developed by Breiman (72), is an ensemble machine learning algorithm consisting of multiple randomized decision trees. This algorithm is able to derive the importance score for each variable *via* statistical permutation tests, both methods correlate adequately (73). Hence, in this study, we calculated the variable importance through the second method using the Gini Index, computed by the equation:


(3) $$\begin{array}{l} VI=\left(X_{j}\right)=\frac{1}{n_{tree}}\left[1-\overset{ntree}{\underset{k=1}{\sum }}Gini{\left(j\right)}^{k}\right] \end{array}$$

where *ntree* is the number of trees.

#### 2.2.6. Classification and Regression Trees

Classification and regression trees (CART) is the name of a family of Decision Tree inference methods that are algorithmically based on either classification or regression. The actual nature of the inference task (classification, regression, clustering-based, or a combination) depends on the type of data available. CART has grown up to be a robust suite of methods, able to deal with mixed data types for which optimized data pre-processing schemes (discretization, normalization, etc.) are available thus expanding the original scope of decision tree inference methods. This algorithm is implemented in the rpart package (74) and uses the Gini Index (as defined by Equation 3) to split each node and allow for optimized feature selection.

#### 2.2.7. C4.5

The machine learning algorithm C4.5 developed by Quinlan (75), builds a decision tree using recursive partitions. Similarly, it applies the gain ratio to select the attribute to split the tree. The gain ratio can be calculated by the following equations:


(4) $$\begin{array}{l} \text{Entropy H}\left(\text{S}\right)=-\overset{\text{m}}{\underset{\text{i}=1}{\sum }}{\text{p}}_{\text{i}}\log_{2}{\text{p}}_{\text{i}} \end{array}$$

where *S* is a set of the data samples distributed on *m* distinct classes, *p*_*i*_ is the probability of samples that belongs to the class.

#### 2.2.8. Extreme Gradient Boosting

The extreme gradient boosting (XGBoost) proposed by Chen and Guestrin (76) is an ensemble machine learning method based on the tree boosting algorithm that can obtain a predictive model with high accuracy and calculates feature importance.

#### 2.2.9. Support Vector Machines

Support vector machines introduced by Bose et al. (77) is a supervised machine learning algorithm. SVM uses mathematical functions (kernels) to take training data as the input space and transform it into an upper dimensional space (feature space), where it aims to obtain a maximum margin hyperplane that divides the data between classes. In this research, we used the linear kernel SVM approach.

#### 2.2.10. Performance Measures

Each model was evaluated using B.ACC, SENS, SPC (78), and G-means performance evaluation metric to determine their predictive performance, customarily defined as follows:


(5) $$\begin{array}{l} SENS=\frac{TP}{TP+FN} \end{array}$$

(6) $$\begin{array}{l} SPC=\frac{TN}{FP+TN} \end{array}$$

(7) $$\begin{array}{l} ACC=\frac{TP+TN}{P+N} \end{array}$$

(8) $$\begin{array}{l} B.ACC=\left(\frac{1}{2}\right)\left(\frac{TP}{P}+\frac{TN}{N}\right) \end{array}$$

(9) $$\begin{array}{l} G-means=\sqrt{SENS*SPC} \end{array}$$

Where *P* = *Positive, N* = *Negative, TP* = *True Positive, FN* = *False Negative, TN* = *True Negative, and FP* = *False Positive*, respectively.

#### 2.2.11. Deep Learning

The basis for improving deep learning is ANN, which works within the association among multiple hidden layers to train and obtain features for the final model (79). The implementation used here is carried out by the library Keras (80) in Python, particularly applying the sequential model; it implies that the ANN is designed by layer.

The input for the network conforms to the number of the established characteristics; then, a convolutional layer is connected with a dimension of 16, after a flattening process is made; the second layer is dense in eight dimensions; finally, a dense network of a single output is obtained; and the activation function is a sigmoid. For the training process, the essential parameters are Adam's optimizer, 2,500 epochs, and a batch size of 100. The selected parameters and architecture are according to proof of better achievement.

## 3. Experimental Setup

The machine learning algorithms were executed using R platform 3.6.1 with RStudio and the following packages: FSelector (81), caret (82), randomForest (83), rpart (84), ROSE (60), performanceEstimation (61), xgboost (85), and Matrix (86). In the case of deep learning, we used the Python programming language.

The computer equipment used was a Workstation Dell, Core Intel(R) Xeon(R) with 32 GB of RAM and 3.50 GHz processor speed, and Windows as an operating system. The computational resources in studies using machine learning applied to help manipulate data about COVID-19 are various and similar to those presented here. Rasheed et al. (87) used 32 GB in RAM with a processor of 3.40 GHz, even when they employed chest images; other works needed a GPU (graphic processor unit) (88). Also, specific studies operate a quantum computer (89), and others utilized fewer resources in processor (2.8 to 3.2 GHz) and RAM (8 or 12 GB) (90–92).

## 4. Results

The first step was to obtain the essential variables of the dataset by applying RF, chi-squared, xgboost, and rpart. Table 2 shows a list of these features sorted in descending order. Similarly, a correlation coefficient analysis was carried out to determine how strong the relationship between the features is.

**Table 2:** Results of the feature selection process.

**RF**	**Chi-squared**	**ANOVA**	**Xgboost**	**rpart**	**Correlation coefficient**	**Consensus set**
BMI	Cocr	Weight	BMI	BMI	Weight	BMI
Waist	Quislt	BMI	Glu	Cocr	Waist	Cocr
Weight	Ovrw	Waist	Cocr	Quislt	BMI	Quislt
Uric	Outli	SBP	HDL	Trig		Uric
Trig		DBP	Quislt	Waist		HDL
HDL		Uric	Trig	HDL		Trig
LDL			Age	EtOH_q		Age
DBP			Slps3	Workf		Glu
Age			LDL	Ovrw		SBP
crea			Crea	Glu		EtOH_q
SBP			SBP	smk		Slps3
Glu			Phyactmet	DBP		
Chol			EtOH_q	Weight		
Height				Uric		

**Figure 2:**
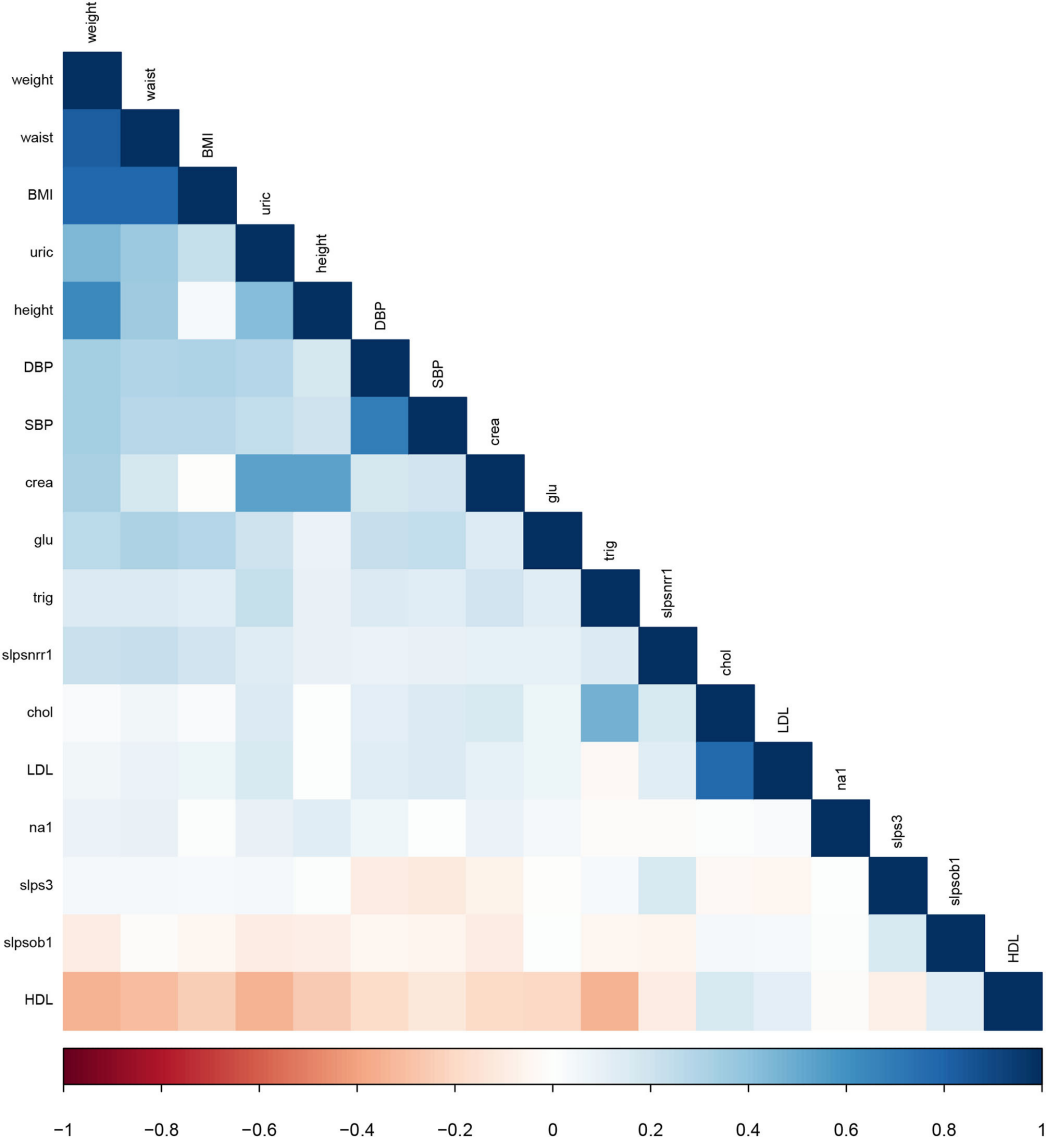
The correlation coefficient of the continuous variables of the dataset.

Figure 2 displays the graphic correlation coefficient of features, where it was possible to identify the statistical dependency structures. The results of this process indicated that weight, waist, BMI, and height were strongly correlated as expected. A fifth subset (*consensus set*) was created by summarizing the effects of the essential variables, comprising the best features obtained by each method; nevertheless, highly correlated variables were eliminated to avoid colinearity.

As shown in Table 2, only the BMI feature remains in the consensus set unlike weight, waist, and uric, which appear in the previous subsets. Each subset of features was tested to find which subset gives the best performance.

In order to choose the best subset of features, we made 30 independent executions using each machine learning algorithm (rpart, C4.5, RF, and SVM) with different seeds, considering the metrics presented in the performance measures section B.ACC is the primary metric to consider. Similarly, it was needed to use balancing methods (SMOTE and ROSE) with each algorithm since a class imbalance in the dataset affected the performance of the algorithms.

In the case of rpart, RF, and SVM, it was necessary to perform a pre-execution for parameter tuning. For tuning RF, the value of the ntree parameter varied between 100 and 1,000, and the mtry was varied between 1 and 10. In all cases, the grid search method introduced by Hsu et al. (93) was applied. Similarly, we used 10-fold cross-validation with ten replays in the training process and ensured the different proofs of the diversity partition of data.

Table 3 displays each classifier's results filtered by applying SMOTE as a balancing method, the average of the 30 executions, and the standard deviation (SD). The highest average result of each classifier in B.ACC is highlighted in bold.

**Table 3:** Feature selection results (SMOTE).

**Classifier**	**Parameters**	**Filter**	**Balancing method**	**B.ACC (%)**	**Sensitivity (%)**	**Specificity (%)**	**G-means (%)**	**PosPred**	**NegPred**
								**Value (%)**	**Value (%)**
rpart	q = 0	RF	Smote	78.68	78.45	78.92	78.62	90.07	60.43
				±2.69	±4.49	±3.71	±0.27	±1.62	±4.86
rpart	q = 0	chi-squared	Smote	78.20	85.98	70.43	70.43	87.68	67.61
				±2.90	±3.07	±5.84	±5.84	±2.05	±4.72
rpart	q = 0	xgbost	Smote	**80.61**	86.91	74.30	80.24	89.26	70.30
				±3.03	±3.27	±6.63	±3.26	±2.41	±4.63
rpart	q = 0	rpart	Smote	79.89	85.74	74.03	79.60	88.98	68.29
				±3.12	±2.94	±5.61	±3.27	±2.16	±4.99
rpart	q = 0	bst	Smote	79.03	87.09	70.97	78.49	88.04	69.48
				±3.63	±2.73	±7.28	± 3.99	±2.61	±4.62
C4.5		RF	Smote	82.13	80.40	83.87	82.10	92.40	63.84
				±1.58	±2.45	±2.43	±1.59	±1.03	±2.85
C4.5		chi-squared	Smote	84.60	90.75	78.44	84.33	91.15	77.80
				±2.25	±1.70	±4.55	±2.41	±1.68	±3.16
C4.5		xgbost	Smote	**85.25**	88.34	82.15	82.15	92.36	74.53
				±2.35	±2.63	±3.81	±3.81	±1.54	±4.36
C4.5		rpart	Smote	83.29	89.80	76.77	82.99	90.42	75.78
				±2.03	±2.46	±3.87	±2.14	±1.42	± 4.16
C4.5		bst	Smote	71.87	72.28	71.47	71.84	68.50	75.08
				±2.60	±3.44	±3.16	±2.61	± 2.73	± 2.70
RF	mtry = 3 ntree = 200	RF	Smote	85.07	83.09	87.04	85.04	93.98	67.94
				±1.05	±1.64	± 0.99	±1.06	± 0.47	±2.20
RF	mtry = 3 ntree = 200	chi-squared	Smote	88.97	93.53	84.41	88.85	93.60	84.37
				± 0.69	±1.34	±1.15	± 0.69	± 0.41	±2.68
RF	mtry = 3 ntree = 200	xgboost	Smote	**90.41**	94.86	85.97	90.30	94.28	87.36
				±1.05	±1.27	±1.75	± 1.07	± 0.67	±2.76
RF	mtry = 3 ntree = 200	rpart	Smote	87.78	92.38	83.17	87.65	93.05	81.88
				± 1.09	±1.55	± 1.88	± 1.10	± 0.71	±3.07
RF	mtry = 3 ntree = 200	bst	Smote	88.85	92.49	85.22	88.77	93.85	82.42
				± 1.16	±1.42	± 1.85	±1.18	±0.73	±2.73
SVM	k = linear c = 1, g = 0.01	RF	Smote	52.12	43.75	60.48	51.35	72.94	30.64
				± 2.37	± 3.53	± 4.42	±2.36	± 2.35	±1.69
SVM	k = linear c = 1, g = 0.01	chi-squared	Smote	69.44	76.47	62.42	69.05	83.21	52.34
				± 1.30	± 3.23	± 2.12	±1.21	± 0.65	±3.05
SVM	k = linear c = 1, g = 0.01	xgboost	Smote	65.45	67.68	63.23	65.39	81.77	44.58
				±1.55	± 2.08	± 2.73	± 1.59	±1.11	± 1.71
SVM	k = linear
c = 1, g = 0.01	rpart	Smote	**72.81**	81.59	64.03	72.27	84.68	58.86
				± 0.93	± 1.41	± 1.54	± 0.96	± 0.55	±1.77
SVM	k = linear
c = 1, g = 0.01	bst	Smote	62.53	63.44	61.61	62.46	80.12	40.93
				±1.42	±2.61	± 3.45	± 1.43	± 1.19	±1.41

*Bold value indicates the highest value achieved by each of the models in the balanced accuracy metric*.

As shown in Table 3 three classifiers got the best performance using the subset generated by Xgboost. Then, RF has the more remarkable achievement in balanced accuracy (B.ACC) of 90.41% and SD of 1.05. Followed by 80.61% in B.ACC and 3.03 in SD using rpart. The third place is the C4.5 model (B.ACC = 85.25% and SD = 2.35). The model obtained by SVM shows a better result through the rpart subset obtained; however, the performance is not the highest; the metrics are B.ACC = 72.81% and SD = 0.93.

Table 4 shows the results using ROSE as a balancing method, where the SVM with the subset of features obtained by rpart achieved the best performance, reaching a B.ACC of 73.11% and SD of 0.0140. Nevertheless, the results obtained with ROSE do not improve in comparison with the results obtained with SMOTE.

**Table 4:** Feature selection results (ROSE).

**Classifier**	**Parameters**	**Filter**	**Balancing method**	**B.ACC. (%)**	**Sensitivity (%)**	**Specificity (%)**	**G-means (%)**	**PosPred**	**NegPred**
							**Value (%)**	**Value (%)**
rpart	q = 0	RF	ROSE	58.03	92.09	23.97	41.65	74.77	72.99
				±4.80	±18.2	±18.0	±14.3	±2.28	±23.2
rpart	q = 0	chi-squared	ROSE	**61.77**	86.40	37.15	55.81	77.18	53.33
				±4.41	±6.22	± 11.68	±6.97	±2.85	±7.13
rpart	q = 0	xgbost	ROSE	60.55	87.77	33.33	53.76	76.26	52.59
				±3.46	±2.18	±7.01	±5.74	±1.86	±6.49
rpart	q = 0	rpart	ROSE	61.43	85.56	37.31	55.66	77.04	51.81
				±4.21	±5.98	±11.38	±7.05	±2.68	±7.07
rpart	q = 0	bst	ROSE	59.77	89.86	29.67	50.45	75.79	56.59
				±3.95	±4.79	±10.68	±9.36	±2.17	±10.80
C4.5		RF	ROSE	57.27	78.04	36.51	48.00	75.17	48.96
				±3.64	±22.74	±21.51	±10.01	±2.30	±14.65
C4.5		chi-squared	ROSE	66.10	85.92	46.29	62.13	79.85	0.5938
				±3.99	±7.71	±12.74	±6.52	±2.99	±8.90
C4.5		xgbost	ROSE	62.44	90.95	33.92	54.95	77.12	63.13
				±2.26	±5.01	±8.60	±5.34	±1.59	±9.19
C4.5		rpart	ROSE	**67.46**	92.56	42.37	62.16	79.72	72.61
				±3.54	±4.92	±8.81	±5.82	±2.08	±11.21
C4.5		bst	ROSE	61.81	91.15	32.47	53.79	76.74	64.11
				±2.44	±5.80	±8.14	±5.58	±1.44	±12.64
RF	mtry = 3
ntree = 200	RF	ROSE	51.65	50.99	52.31	48.73	71.70	31.38
				±4.31	±18.89	±15.62	±6.30	±4.01	±4.80
RF	mtry = 3 ntree = 200	chi-squared	ROSE	**65.43**	83.93	46.94	60.89	79.77	61.04
				±4.16	±12.29	±15.95	±8.47	±3.07	±14.25
RF	mtry = 3 ntree = 200	xgboost	ROSE	64.33	94.08	34.58	56.92	58.97	85.57
				±1.91	±1.74	±4.19	±3.24	±2.72	±3.23
RF	mtry = 3 ntree = 200	rpart	ROSE	64.66	92.23	37.08	58.38	59.43	82.85
				±1.81	±2.05	±4.17	±2.95	±2.74	±3.47
RF	mtry = 3 ntree = 200	bst	ROSE	64.56	93.78	35.34	57.42	59.19	85.30
				±2.12	±2.18	±4.88	±3.56	±2.93	±3.69
SVM	k = linear c = 1, g = 0.01	RF	ROSE	57.55	58.28	56.83	57.10	76.71	36.18
				±2.83	±8.20	±7.42	±2.94	±2.08	±3.26
SVM	k = linear c = 1, g = 0.01	chi-squared	ROSE	68.97	78.32	59.62	68.02	82.64	53.83
				±1.86	±6.32	±7.16	±2.50	±1.60	±4.70
SVM	k = linear c = 1, g = 0.01	xgboost	ROSE	65.47	68.52	62.42	65.40	81.68	45.19
				±1.49	±5.59	±5.05	±5.31	±1.17	±2.93
SVM	k = linear c = 1, g = 0.01	rpart	ROSE	**73.11**	79.93	66.29	72.63	85.32	58.08
				±1.40	±4.74	±5.36	±1.58	±1.45	±4.23
SVM	k = linear c = 1, g = 0.01	bst	ROSE	65.54	69.03	62.04	65.20	81.67	45.51
				±1.45	±6.03	±5.80	±1.45	±1.36	±3.17
Deep learning		RF		61.52	24.02	99.01	47.83	55.60	77.82
				± 2.53	±5.46	± 6.00	± 9.55	± 4.38	± 1.11
Deep learning		chi-squared		61.35	31.26	91.45	53.31	57.90	78.17
				±2.08	±4.65	±1.86	±3.59	±5.17	±1.08
Deep learning		xgboost		63.19	30.86	95.53	54.22	73.03	78.80
				±1.42	±2.91	±2.05	±2.41	±7.85	±6.57
Deep learning		rpart		**65.80**	34.36	97.39	57.77	83.10	79.97
				±1.69	±3.56	±6.05	±2.99	±3.06	±8.35
Deep learning		bst		63.89	29.77	98.01	53.85	84.72	78.97
				± 2.31	±4.59	±0.77	±4.25	±5.16	±1.08

*Bold value indicates the highest value achieved by each of the models in the balanced accuracy metric*.

The worst performance was obtained by the deep learning model since the sensitivity is low (refer to Table 4), which may be due to the number of existing patient records since the capacity of neural networks with a more significant amount of data has been demonstrated. Therefore, according to the metrics results obtained by the machine learning classifiers, it was feasible to determine the most suitable model and the main characteristics of participants who contracted COVID-19.

The finest model was RF with a ntree of 200 and a mtry of 3, and the subsequent attributes obtained by Xgboost: BMI, glu, cocr, HDL, quislt, trig, age, slps3, LDL, crea, SBP, phyactmet, EtOH_q, and weight. These relevant features are firmly related to COVID-19 infections, such as the consumption of alcoholic drinks (94–96), sleep disorders (97), BMI (98–100), age (101, 102), and physical activity (103).

## 5. Discussion

It is interesting to notice that even though computational intelligence and machine learning approaches at the level presented here are not able to provide any mechanistic nor semi-mechanistic explanation of the underlying phenomena behind their predictions; since this is not the goal for which they were designed.

These tools can be used however to perform timely predictions based on the data. These predictive models can thus be used by decision makers and public health authorities for the design and implementation of policy and actionable measures that are especially needed in critical times such as the ones presented by the global COVID-19 pandemic.

Hence, even though it is quite likely that the selected features are indeed *proxies* for the actual (unknown and likely unmeasured) determinants of infection; they present an important opportunity since many of them are *actionable* (either controllable or measurable).

Take for instance the selected features in the *Consensus set*. As presented in Table 2, the set consists of 11 features: body mass index (measurable and to some extent controllable), worry for COVID-19 contagion (measurable, or more properly, surveyable and to a certain extent controllable), isolation during the COVID-19 pandemic (measurable and controllable), uric acid levels (measurable and to some extent controllable), HDL levels (measurable and to some extent controllable), triglycerides levels (measurable and to some extent controllable), age (measurable), glucose levels (measurable and to some extent controllable), Systolic Blood Pressure (measurable and to some extent controllable), frequency of alcohol consumption during the pandemic (surveyable and controllable), and sleep somnolence (surveyable). Similar remarks can be made about most other features selected by the diverse approaches used in this study.

Several of these features have been of course analyzed in the context of disease severity, *once the individuals are already infected*, but the role they may be playing or their potential associations with the infection itself, have been less discussed in the literature with some remarkable exceptions regarding BMI (104, 105), HDL (106, 107), age (108), alcohol consumption (109), and somnolence (110), among others.

In a nutshell, even if the actual risk factors are not the features selected by our machine learning algorithm, these are likely either a *combination* of those features selected or a statistically dependent set of these. In either case, it is likely that by controlling/modifying these issues, COVID-19 infections may become intervened. Hence, knowing these variables, that in the end predicted with very high sensitivity and specificity COVID-19 infections in an urban population of a large metropolitan area such as Mexico City, may provide some opportunities for interventional policy.

A number of these featured variables for instance are related to metabolism, food consumption, exercise habits, and lifestyle. Though these issues are not easily modifiable in the short run, public health interventions can be made to address them in a medium to a long time.

However, since these indicators are measurable or surveyable, this opens the possibility to implement policy measures to protect *high risk* individuals (HRIs). For instance, HRIs can be prioritized to work from home or they can be tested more often, etc. Indeed, the data-driven design of non-pharmaceutical interventions to alleviate the burden caused by COVID-19 infections has been discussed recently in diverse contexts including social contact structure, human mobility, and environmental constraints (24–27).

In fact, in recent times, it has been consistently discussed how machine learning approaches may be extremely valuable tools for the design of public health policy (111, 112). This is particularly true for the management of infectious diseases, both in the clinical decision and primary care (113, 114), epidemiological surveillance (115), social perception (116), and policy making levels (117–119).

In particular, feature selection approaches to risk assessment of infectious diseases have been successfully applied in the case of tuberculosis (120), zika (121), dengue (122), clostridium difficile (123), HIV (124), and even COVID-19 (125, 126). These previous efforts have shown the advantages of these approaches as reliable tools for epidemic outbreak prevention and containment.

In the particular case of the present study, we can highlight the fact that the features selected are not only measurable/surveyable but are actually relatively easy to measure. Indeed, measurements and surveys are low cost, easily manageable, and highly scalable. These characteristics are relevant in the context of the actual implementation of the predictive models here presented to design policy and implement actions to tackle a challenging situation such as the COVID-19 pandemic.

## 6. Conclusion

Machine learning algorithms have played a critical role in the diagnostics and containment of the COVID-19 pandemic since, through multivariate methods, these tools may provide an overview of the association between various factors and their relationship regarding potential risk factors for infection, unveiling hidden patterns that may result essential for the proper implementation of public health policy.

In the approach followed in this study, we have implemented and bench-marked several state of the art feature selection methods on a dataset obtained in real time over a well-studied cohort consisting of adults of both sexes living in the metropolitan area of Mexico City. We believe that some of our results, aside from being useful in our socio-geographical context, maybe somehow generalizable to other similar urban populations.

We are confident that embracing data-driven policy designs may further contribute to faster, targeted interventions to cope with current and future challenges to public health, such as the case of the COVID-19 pandemic.

## Data Availability Statement

Due to personal privacy issues, raw data cannot be provided unless proper inter-institutional ethico-legal agreements are signed. Anonymized data is available upon request. For data related enquiries please contact Dr. Mireya Martinez-Garcia, mireya.martinez@cardiologia.org.mx.

## Author Contributions

TR-d implemented computing code and algorithmics and contributed to drafting the manuscript. MM-G performed clinical, sociomedical, and health policy research and contributed to drafting the manuscript. MF-M performed the clinical assessment. LL-T contributed to clinical assessment. GG-E designed computational strategy, implemented computing code and algorithmics, evaluated performance measures, co-supervised the project, and drafted the manuscript. EH-L devised the strategy, co-supervised the project, performed the technical assessment, and revised and edited the manuscript. All authors read and approved the submitted version of the manuscript.

## Funding

This research was supported by the National Council of Science and Technology (CONACYT, México), Cátedras CONACYT 1591.

## Conflict of Interest

The authors declare that the research was conducted in the absence of any commercial or financial relationships that could be construed as a potential conflict of interest.

## Publisher's Note

All claims expressed in this article are solely those of the authors and do not necessarily represent those of their affiliated organizations, or those of the publisher, the editors and the reviewers. Any product that may be evaluated in this article, or claim that may be made by its manufacturer, is not guaranteed or endorsed by the publisher.
